# The validation of a three-stage screening methodology for detecting active convulsive epilepsy in population-based studies in health and demographic surveillance systems

**DOI:** 10.1186/1742-7622-9-8

**Published:** 2012-11-21

**Authors:** Anthony K Ngugi, Christian Bottomley, Eddie Chengo, Martha Z Kombe, Michael Kazungu, Evasius Bauni, Caroline K Mbuba, Immo Kleinschmidt, Charles R Newton

**Affiliations:** 1 The Centre for Geographic Medicine Research – Coast, KEMRI/Wellcome Trust Research Programme, Kilifi, Kenya; 2 Department of Infectious Disease Epidemiology, Faculty of Epidemiology and Population Health, London School of Hygiene and Tropical Medicine, London, UK; 3 Studies of Epidemiology of Epilepsy in Demographic Surveillance Systems (SEEDS) – INDEPTH Network, Accra, Ghana; 4 MRC Tropical Epidemiology Group, Faculty of Epidemiology and Population Health, London School of Hygiene and Tropical Medicine, London, UK; 5 INDEPTH Network, Accra, Ghana; 6 Clinical Research Unit, London School of Hygiene and Tropical Medicine, London, UK; 7 Neurosciences Unit, UCL Institute of Child Health, London, UK; 8 Department of Psychiatry, University of Oxford, Oxford, UK

**Keywords:** Epilepsy, Three-stage methodology, Screening, Validation, Sensitivity, LMIC

## Abstract

**Background:**

There are few studies on the epidemiology of epilepsy in large populations in Low and Middle Income Countries (LMIC). Most studies in these regions use two-stage population-based screening surveys, which are time-consuming and costly to implement in large populations required to generate accurate estimates. We examined the sensitivity and specificity of a three-stage cross-sectional screening methodology in detecting active convulsive epilepsy (ACE), which can be embedded within on-going census of demographic surveillance systems.

We validated a three-stage cross-sectional screening methodology on a randomly selected sample of participants of a three-stage prevalence survey of epilepsy. Diagnosis of ACE by an experienced clinician was used as ‘gold standard’. We further compared the expenditure of this method with the standard two-stage methodology.

**Results:**

We screened 4442 subjects in the validation and identified 35 cases of ACE. Of these, 18 were identified as false negatives, most of whom (15/18) were missed in the first stage and a few (3/18) in the second stage of the three-stage screening. Overall, this methodology had a sensitivity of 48.6% and a specificity of 100%. It was 37% cheaper than a two-stage survey.

**Conclusion:**

This was the first study to evaluate the performance of a multi-stage screening methodology used to detect epilepsy in demographic surveillance sites. This method had poor sensitivity attributed mainly to stigma-related non-response in the first stage. This method needs to take into consideration the poor sensitivity and the savings in expenditure and time as well as validation in target populations. Our findings suggest the need for continued efforts to develop and improve case-ascertainment methods in population-based epidemiological studies of epilepsy in LMIC.

## Introduction

Epilepsy is a common non-communicable neurological condition and a significant cause of disability and mortality [1]. It is estimated to affect nearly 70 million people worldwide, 90% of who live in low and middle income countries (LMIC) [2]. There is a wide range of prevalence estimates from studies estimates in LMIC [2]. It is unclear if this heterogeneity is caused by different methods and/or tools used, many of which have not been validated in the target populations [3-5].

In high income countries (HIC), researchers utilize medical and service records to provide epidemiological data on epilepsy. In LMIC this methodology is unreliable since there is low usage and/or lack of access to health care facilities by majority of the population and where available (e.g. in urban settings) medical records and diagnostic facilities are often poor [6-10]. Thus single round surveys (done by non-medical field personnel) and key informants have been used but these under-report the prevalence [11]. Two-stage surveys in which the population is screened with a questionnaire, with the diagnoses confirmed by clinical evaluation are recommended [12] and have been used in several studies [13-21]. However, this method is costly to implement in large populations since the first stage takes considerable time and the second stage requires qualified medical personnel who often have to assess a large number of false positives. With a condition such as epilepsy, there are no simple diagnostic tests (e.g. blood measurements) and the diagnosis depends upon history and assessment by experienced specialists. There are relatively few specialists in LMIC and they are expensive to employ for epidemiological surveys [22,23]. Epidemiological studies are often conducted on high-risk populations (e.g. those with a high prevalence of putative risk factors) [24-26]. These may generate high estimates of prevalence that are not representative of wider populations [27]. In these resource-limited settings, identification of patients with epilepsy is further compounded by stigma-related concealment of patients by their families and/or their symptoms.

These methodological problems may have led to under ascertainment of epilepsy cases in studies conducted in LMIC, with the inevitable consequence of incomplete coverage of target populations for public health interventions. Thus, there is need to survey larger populations to provide robust estimates of distribution using methods that maximise detection of cases with least cost and effort [11].

To address these issues in epidemiological studies of epilepsy, we have used a three-stage methodology to screen large populations within Health and Demographic Surveillance Systems (HDSS) [28], under the INDEPTH network (http://www.indepth-network.org/). Within the HDSS, censuses are conducted 1–3 times a year, in which non-medical personnel conduct re-enumeration and vital status registration by interviewing a senior member of the household. Thus, the screen for convulsions (first stage of the three-stage methodology) needs to be embedded within on-going HDSS census to be administered to the entire population to minimise cost. In this paper, we determine the sensitivity and specificity of the three-stage method in detecting active convulsive epilepsy (ACE) in population-based studies and compare the expenditure with that of a two-stage survey.

## Methods

### The study setting and study population

The study was conducted within the Kilifi HDSS (KHDSS - http://www.kemri-wellcome.org/khdss/) in the Coast province of Kenya. The study area covers 891 square kilometers in 15 administrative locations with 40 sub-locations. Demographic surveillance and vital registration are performed 2–3 times a year.

The residents are mainly Mijikenda, a Bantu grouping of nine tribes with Giriama (45%), Chonyi (33%) and Kauma (11%) being most common. About 55% of the population is considered absolutely poor, per capita income ~ USD 10 per month. The majority (80%) are subsistence farmers and literacy levels are low (about 45%).

### Description of the three-stage cross-sectional survey method (prevalence survey)

#### Stage 1 (SI): the general population screen

The first stage screens the entire resident population using a simple two-item tool to detect convulsions (Additional file 1), which is administered to a senior member of each household (as a proxy for members of that household) by a non-medical fieldworker. The questions were piloted to maximise their sensitivity to detect individuals with the main symptoms of convulsive epilepsy (i.e. convulsions). This stage uses HDSS census field staff to screen populations rapidly during their routine re-enumeration and vital registration.

#### Stage 2 (SII): the condition-specific screen

People identified with a history of convulsions in SI were followed-up within a week by different interviewers with more extensive training in epilepsy who administered a more detailed and specific questionnaire to the individual or their care givers (if they were minors or cognitively challenged) to identify possible cases. This tool was based on that used in other studies [12] (Additional file 2). This stage aimed to reduce the number of false positives by using a tool with higher specificity than that used in the first stage in order to reduce the cost of diagnosis in the subsequent stage.

#### Stage 3 (SIII): Confirmation of diagnosis

The respondents identified as potential cases of ACE in SII were invited for clinical assessments to confirm the diagnosis within 1–2 weeks of SII. The diagnosis of ACE was based upon a detailed clinical history taken by an experienced clinician. ACE was defined as at least two unprovoked convulsions (tonic and/or clonic seizures), of which one occurred within 12 months of the clinical assessment [28,29].

The three-stage survey method identified an individual as a case of ACE if they were positive in all the three stages. This three-stage survey method was tested in this validation study.

### Assessment of the three-stage methodology using a survey conducted by clinicians

#### Sampling and follow-up of the validation sample

A random sample for the validation of the three-stage survey methodology was selected from the 2008 Kilifi HDSS population database using the ‘RAND()’ command in MySQL Version 5 open source database (Oracle Corporation, Redwood Shores, CA, USA). A sample size of 5796 was estimated to determine the sensitivity of the three-stage method (assumed to be 85%) with a precision of 13% (i.e., half the width of the 95% Confidence Interval (95%CI)) [30] in a population with a prevalence ~ 5/1000 [28]. This sample was interviewed in the three stages of the prevalence survey in 2008 as described above.

#### Clinical survey (Validation Survey)

To validate the three-stage methodology, everyone in the random sample had the SII questions administered by non-medical fieldworkers (independent of their status in SI of the three-stage methodology), as well as being interviewed by experienced clinicians with the proforma administered in the SIII of the prevalence survey (this was the Clinical Survey or the “gold standard” for validating the three-stage method (‘test’) described above). This validation was conducted after the prevalence survey from May 2009 to April 2011. Incident cases (those negative on the prevalence survey and positive in the Clinical Survey but who developed ACE after the three-stage prevalence survey) were treated as true negatives since they were negative during the prevalence survey. SIII was conducted twice on those that were positive in SII of the prevalence survey (i.e. within the prevalence survey and the validation/Clinical Survey – because SIII was the Clinical Survey/”gold standard”) and subjects were classified positive for ACE if they were positive in either of the two SIII assessments.

### Analysis

#### Estimation of Sensitivity

Sensitivities were estimated as: *TP/(TP+FN)*, where TP = True Positive (i.e., positive on prevalence survey and Clinical Survey) and FN = False Negative (i.e., negative on prevalence survey but positive on Clinical Survey). The sensitivities of SI (single stage) and (SI & SII) (two-stage) and the (SI & SII & SIII) (three-stage) methods were evaluated against the Clinical Survey. For example, the sensitivity of the three-stage method was the proportion of individuals who were positive in all stages (SI+, SII+, SIII+) in the prevalence survey among those identified as cases of ACE in the Clinical Survey.

#### Estimation of Specificity

Specificity was estimated as: *TN/(TN+FP)*, where TN = True Negative (i.e., those negative in both the prevalence and Clinical Surveys) and FP = False positive (positive in the prevalence survey but negative in the Clinical Survey). The specificities of single, two and three-stage methods were estimated. For the three-stage method the specificity is 100% since there are no false positives (because SIII of the prevalence survey is the Clinical Survey). For the single and two-stage methods, specificity may be less than 100%. In addition we estimated the proportions of false positives (1-specificity) and false negatives (1-sensitivity).

#### Expenditure comparison study

We compared the financial expenditure of a two-stage survey (in which a clinical diagnosis is made by experienced clinicians on those identified as SI positive by lay field personnel) and the current three-stage epilepsy survey. Our analyses were based on the SI and SII positive proportions and the marginal expenditure incurred in the three-stage survey of 2008. In both situations, we assumed that the first stage (SI) would not incur any expenditure since it is embedded within on-going census. For the two-stage survey, SII would be the definitive stage since it is conducted by clinicians using the clinical history tool used in SIII of the three-stage survey.

All analyses were performed in STATA version 11 (StataCorp, College Station, TX, USA).

### Ethical considerations

The study protocol was explained to all participants by the interviewing clinicians. Written informed consent was obtained from all participants or their caregivers if they were less than 18 years of age. The study was approved by the Kenya Medical Research Institute/ National Ethical Review Committee.

## Results

### Flow of validation subjects in the Clinical Survey

**Figure 1:**
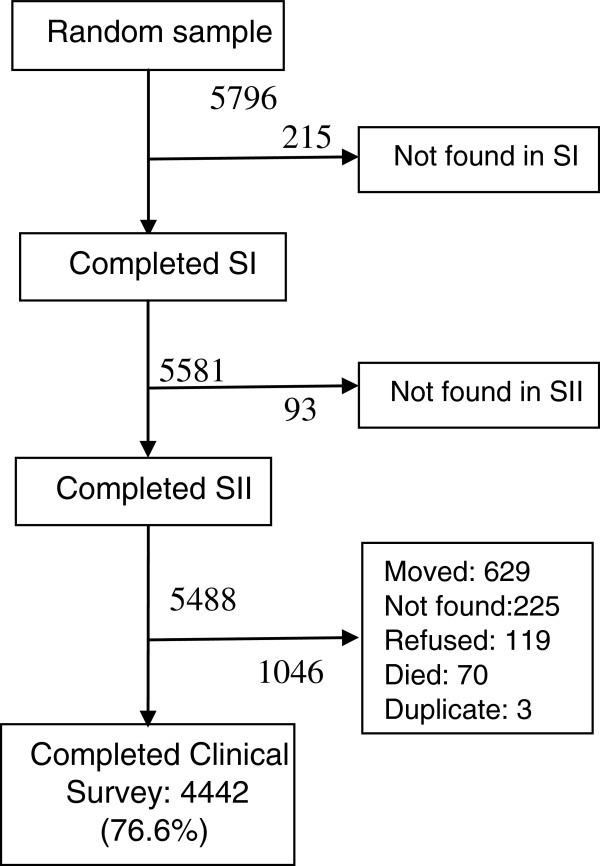
Flow of subjects in the Clinical Survey.

The Clinical Survey targeted a sample of 5488 participants who completed both SI and SII of the prevalence survey. Of these, 1046 (19.1%) were lost to follow-up: 629 (60.1%) had moved, 225 (21.5%) could not be traced, 119 (11.4%) refused consent and 70 (6.7%) had died, while 3 (0.3%) were found to have been duplicate records (Figure 1). The reasons for loss to follow-up were ascertained from the regularly updated HDSS population and vital events register.

**Figure 2:**
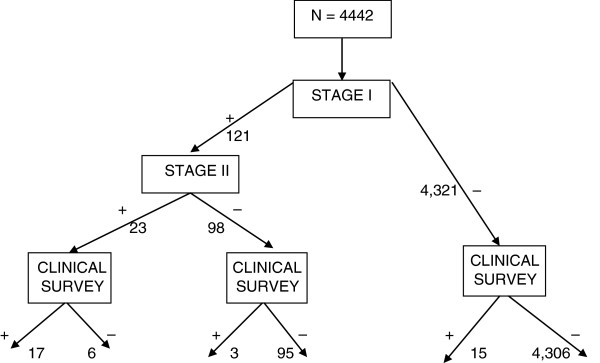
Response status of subjects within the prevalence and Clinical Surveys.

Of 4442 subjects screened in the Clinical Survey, 18 ACE cases identified during the Clinical Survey were negative in the prevalence survey i.e. the 4321 (SI-) and 98 (SI+, SII-) (Figure 2). The reasons for the 18 false negatives were: i) 15 were negative in SI (reported no history of convulsions) but the Clinical Survey documented that they had convulsions which occurred before the prevalence survey and therefore should have been identified as potential cases; ii) the remaining 3 subjects were positive in SI but were classified as febrile convulsions in SII. We interviewed 13 of the 15 false negatives (negative in SI) with a stigma scale [31]. All the 13 respondents had high perceived stigma (based on a 66^th^ percentile cut-off in the same epilepsy population) compared to the other cases of ACE of whom only 33% felt stigmatized [31].

### Sensitivity and Specificity

Sensitivity decreased and specificity increased with the number of stages. The three-stage method (SI & SII &SIII) had sensitivity of 48.6 % but with a specificity of 100.0 % (Table 1). Sensitivity was highest for the one-stage (SI) screening method (57.1 %), but it also had the lowest specificity (97.7 %). Sensitivity and specificity estimates of combinations of various stages within the three-stage methodology are displayed in Table 1.

**Table 1:** Estimation of the sensitivity and specificity of the single- and multi-stage survey methodologies using the Clinical Survey as gold standard

**Method**	**Sensitivity, 95% CI**		**Specificity, 95% CI**		**TP**	**FN**	**FP**	**TN**
**One-stage (SI)**	57.1	39.4 – 73.7	97.7	97.2 – 98.1	20	15	101	4306
**Two-stage (SI & SII)**	51.4	32.4 – 67.6	99.9	99.7 – 100.0	18	17	6	4401
**Three-stage (SI & SII & SIII)**	48.6	31.4 – 66.0	100.0	–	^†^ 17	18	^†^ 0	4407

TP=true positive; FP=false positive; FN=false negative; TN=true negative.

^†^TP and FP are derived from 2^nd^ SIII/Clinical Survey on those that were SII+ (i.e. SIII was done twice on the SII+ participants).

### Expenditure comparison study

From the proportion of respondents who were positive in SI (2.2%), we estimated that we would require to follow-up 6183 people in SII of the study. In the three-stage method (where SII is conducted by lay field personnel), this would take 29.4 months to complete at a cost of USD 109,120 (Table 2). In the two-stage method (in which SII is conducted by clinicians), screening the 6183 people positive in SI would take 42.1 months and cost USD 171,922, which is 1.6 times more expensive than the three-stage method (Table 3).

**Table 2:** Estimates of the expenditure of the three-stage survey conducted by lay field personnel

**Stage II field work**					
**SI +ve rate (%)**	2.2				
**SI Population**	233,881				
		**Duration†**		**Expenditure**	
	**Follow-up**	**FW days**	**FW months**	**Salary (Ksh)**	**Salary (USD)**
**No of SI cases**	5152.4	515.2	24.5	613,381	7667
20% Follow-up*	1030.5	103	4.9	122,676	154
**Sub-total 1**	6182.9	618.3	29.4	736,057	9201
**Stage II transport costs**					
		**Duration**		**Costs**	
	**Follow-up**	**FW days**	**FW months**	**Cost (Ksh)**	**Cost (USD)**
**No cases followed up**	6182.9	618.3	29.4	7,419,454	92,743
**Sub-total 2**		618.3	29.4	7,419,454	92,743
**Stage II cost**				8,155,511	101,935
**Stage III**					
**SII +ve rate (%)**	21.8				
**No of SII cases**	1125.1				
		**Duration†**		**Costs**	
	**No screened**	**Clinic days**	**Clinic months**	**Salary (Ksh)**	**Salary (USD)**
**No of SII +ve cases**	1125.1	160.7	7.7	574,019	7175
**No screened in SII**	1125.1	160.7	7.7	574,019	7175
**Total Expenditure**				**8,729,530**	**109,119**

*Assume that at least 20% of the SI positive cases will be followed up in the field at least twice.

† Both SII and SIII run concurrently therefore the total duration is 29.4 months.

1) One field worker interviews 10 participants per day on average.

2) One working month = 21 working days.

3) A fieldworker's salary is Ksh 25,000 per month.

4) The study clinician interviews/assesses 7 participants per day.

5) A clinician's salary is Ksh 75,000 per month.

6) 1 USD = 80 Ksh.

7) FW = Field work.

8) Transport costs:

a) Distance/day: estimated at 200km.

b) Cost per = Ksh 60/km (vehicle).

**Table 3:** Estimates of the expenditure of the two-stage survey conducted by clinicians

**Stage II field work**					
**SI +ve rate (%)**	2.2				
**SI Population**	233,881				
		**Duration**		**Expenditure**	
	**Follow-up**	**FW days**	**FW months**	**Salary (Ksh)**	**Salary (USD)**
**No of SI cases**	5152.4	736.1	35.1	2,628,775	32,860
20% Follow-up*	1030.5	147.2	7	525,755	6572
**Sub-total 1**	6182.9	883.3	42.1	3,154,530	39,432
**Stage II transport costs**					
		**Duration**		**Costs**	
	**Follow-up**	**FW days**	**FW months**	**Cost (Ksh)**	**Cost (USD)**
**No cases followed up**	6182.9	883.3	42.1	10,599,220	132,490
**Sub-total 2**		883.3	42.1	10,599,220	132,490
**Total Expenditure**				**13,753,749**	**171,922**

*Assume that at least 20% of the SI positive cases will be followed up in the field at least twice.

1) One field worker interviews 10 participants per day on average.

2) One working month = 21 working days.

3) A fieldworker's salary is Ksh 25,000 per month.

4) The study clinician interviews/assesses 7 participants per day.

5) A clinician's salary is Ksh 75,000 per month.

6) 1 USD = 80 Ksh.

7) FW = Field work.

8) Transport costs:

a) Distance/day: estimated at 200km.

b) Cost per = Ksh 60/km (vehicle).

## Discussion

The three stage method had a low sensitivity of 48.6%, but cost 63% of the two stage survey. Sensitivities were slightly higher for the one-stage (57.1%) and two-stage (51.4%) methods. Specificities were marginally lower in single-stage (97.7%) compared to the three-stage method (100%). It would be important to improve the sensitivity of the questions in stage I in future work. However, estimates of prevalence obtained using the current three stage method can be adjusted to allow for the low sensitivity where this method is likely to be useful due to logistical and cost considerations e.g. in HDSS sites.

In this study, we examined a population-based three-stage system for detecting ACE (where a subject was classified as a case if they were positive in all the three stages). Other studies have validated only hospital-based survey methods [17,20,32] or determined the validity of a screening tool (within a three-stage method) in detecting epileptic seizures but not epilepsy [12]. Unlike the validation of population-based studies, generalization of validation parameters of hospital-based studies is limited by selection bias (due to overrepresentation of cases with more severe forms of epilepsy). Additionally, people who lack knowledge of epilepsy may fail to seek treatment and therefore are not captured in hospital-based validations. Other limitations in hospital-based validation studies are inadequate sample sizes and they may not represent the field situation. Thus these studies therefore provide less accurate (often inflated) estimates of sensitivity than population-based validation [12]. The validation of population-based methods is applied directly to a wider population, hence the findings are more generalizable, although this is usually costly. Furthermore, compared to validation of population-based studies, the validity of hospital-based studies may not be influenced by stigma-related concealment of seizures.

The precision of the validation depends upon the sample size, which for our study was statistically determined and was larger than for other validation studies [17,32,33]. In our study, the number of false positives was highest in SI; since the SI questions targeted all (including febrile) convulsions. Even a small proportion of false positives in a large population would have considerable logistical and cost implications. For instance, 2.3% of false positives among this study population of 233,880 individuals results to 5379 false positive individuals that would otherwise have to be screened in SIII of the prevalence survey. Inclusion of SII substantially reduces the false positives screened in SIII, which in our study declined from 2.3% to 0.1%.

We found that multi-stage methods (SI&SII and SI&SII&SIII) had poorer sensitivity than SI alone. This was observed in another validation study where (like in our study) if only epilepsy specific questions were used, sensitivity was substantially lower (and the specificity higher) than when questions on epilepsy and other seizures were used [33,34]. These observations suggest that questions about seizures under any circumstances are important in avoiding false negatives.

In our study, stigma-related non-response could be the main cause of the low sensitivity of the three-stage method. This is suggested by the moderate to high perceived stigma scores for all the false negatives compared to the epilepsy population (those identified in the prevalence survey) in which only 33% felt stigmatized [31]. Stage I (which contributed the largest proportion of false negatives i.e. 15/18) was conducted by HDSS field staff who are usually resident in the study community and who were also involved in the routine re-enumerations within the study area. The sensitivity of SI may also depend on the cultural setting (e.g. SI screening questions might be more frequently misinterpreted in communities with low literacy levels) and the skills of the field staff in administering sensitive questions (pertaining to a stigmatized condition). For example, the percentage of false negatives was much lower (23.4%) in an Australian when a screening questionnaire was administered to study known (physician diagnosed) epilepsy cases [35]. In contrast, the clinical survey (our “gold standard”) was conducted by clinicians experienced in the diagnosis of epilepsy, of whom individuals with epilepsy or their guardians may have been more trusting and/or have expected benefits such as treatment or advise. A high sensitivity (79.3%) was estimated in another field validation in which rural doctors conducted an equivalent of SII of our prevalence survey [12]. However, it is difficult to compare our validation with this study due to differences in case definitions and sources of cases [12,17].

A lack of awareness of convulsions in family members by the household heads could also have led to the low sensitivity of SI. Household heads (or their spouses) were the primary respondents in the HDSS re-enumeration. Individuals identified as having convulsions by the household head in SI (or their caretakers if they were children or cognitively impaired) were interviewed in the subsequent survey stages and in the Clinical Survey.

Three cases of epilepsy were lost in the prevalence survey between SI and SII because field personnel who conducted SII were unable to distinguish between febrile and non-febrile convulsions. The sensitivity of the three-stage method could therefore be improved by better training the SII field staff to distinguish between different types of convulsions.

A two-stage epilepsy survey conducted by clinicians would be more expensive and of longer duration than a three-stage survey in which the initial stages are carried out by non-medical field personnel. This is because medically trained personnel require higher salaries and lack the field-work experience of lay field workers. However, for this additional expenditure, the improvement in sensitivity would be limited. Only false negatives in SII would be eliminated and so the sensitivity would at best be equivalent to the sensitivity of SI.

### Limitations of the study

A major limitation of this study was the attrition of subjects over the two years of the Clinical Survey. These losses were observed despite efforts to locate individuals. There were, however, no differences in age, sex and SI status (either positive or negative in SI of the three-stage screening) between those lost and those that completed the study.

There could have been an increase in awareness of epilepsy in this population following the three-stage prevalence survey. This could have reduced stigma and increased knowledge of epilepsy, yielding more positive responses within the Clinical Survey, since the latter survey was conducted over a 2 year period after the prevalence survey. This hypothesis is supported by a study conducted on the same population after the prevalence survey which reported only moderate levels of perceived stigma [31].

## Conclusion

There is an urgent need to obtain accurate assessment of the burden of neuropsychiatric conditions in regions of the world where it is difficult to obtain statistics. We have shown that a three-stage method can be used to screen large populations for these conditions during a census in an efficient and cost-effective manner, provided the sensitivity of first stage questions is high or the loss of sensitivity at this stage can be adjusted. Stigma-related concealment of potential cases could be decreased with community sensitization and education.

## Competing interests

The authors declared that they have no competing interest.

## Authors’ contributions

This study was conceived by AKN and CRN. AKN wrote the study protocol with input from CB, IK, CRN, CKM and EB. AKN managed data collection, which was done by EC, MZK and MK. AKN analyzed the data with input from CB, IK and CRN. AKN wrote the first draft. All authors reviewed all drafts and approved the final submitted manuscript.

## Supplementary Material

Additional file 1 Stage I (SI) of the cross-sectional survey: (Census screen for convulsions). Click here for file

Additional file 2 Stage II (SII) screening questions. Click here for file

## References

[B1] WHO Epilepsy in the WHO Africa region, Bridging the Gap: The Global campaign against epilepsy "Out of the Shadows" 2004 Geneva: WHO

[B2] Ngugi AK Bottomley C Kleinschmidt I Sander JW Newton CR Estimation of the burden of active and life-time epilepsy: a meta-analytic approach Epilepsia 2010 51 5 883 890 10.1111/j.1528-1167.2009.02481.x 20067507 PMC3410521

[B3] Sander JW Shorvon SD Epidemiology of the epilepsies J Neurol Neurosurg Psychiatry 1996 61 5 433 443 10.1136/jnnp.61.5.433 8965090 PMC1074036

[B4] Leonardi M Ustan TB The global burden of epilepsy Epilepsia 2002 43 Suppl 6 21 25 12190974 10.1046/j.1528-1157.43.s.6.11.x

[B5] Sander JW The epidemiology of the Epilepsies Current Opin Neurol 2003 16 165 170 10.1097/00019052-200304000-00008 12644744

[B6] Alam N Chowdhury HR Bhuiyan MA Streatfield PK Causes of death of adults and elderly and healthcare-seeking before death in rural Bangladesh J Health Popul Nutr 2010 28 5 520 528 20941904 10.3329/jhpn.v28i5.6161 PMC2963775

[B7] Olusanya BO Alakija OP Inem VA Non-uptake of facility-based maternity services in an inner-city community in Lagos, Nigeria: an observational study J Biosoc Sc 2010 42 3 341 358 10.1017/S0021932009990526 19951456

[B8] Mesfin MM Newell JN Walley JD Gessessew A Madeley RJ Delayed consultation among pulmonary tuberculosis patients: a cross sectional study of 10 DOTS districts of Ethiopia BMC Public Health 2009 9 53 10.1186/1471-2458-9-53 19203378 PMC2647537

[B9] Shaikh BT Hatcher J Health seeking behaviour and health service utilization in Pakistan: challenging the policy makers J Public Health (Oxf) 2005 27 1 49 54 10.1093/pubmed/fdh207 15590705

[B10] Bhatia JC Cleland J Health-care seeking and expenditure by young Indian mothers in the public and private sectors Health Policy Plan 2001 16 1 55 61 10.1093/heapol/16.1.55 11238431

[B11] Kaamugisha J Feksi AT Determining the prevalence of epilepsy in the semi-urban population of Nakuru, Kenya, comparing two independent methods not apparently used before in epilepsy studies Neuroepidemiology 1988 7 115 121 10.1159/000110144 3136404

[B12] Placencia M Sander JW Shorvon SD Ellison RH Cascante SM Validation of a screening questionnaire for the detection of epileptic seizures in epidemiological studies Brain 1992 115 783 794 10.1093/brain/115.3.783 1628202

[B13] Osuntokun BO Adeuja AO Nottidge VA Bademosi O Olumide A Ige O Yaria F Bolis CL Schoenberg BS Prevalence of the epilepsies in Nigerian Africans: a community-based study Epilepsia 1987 28 3 272 279 10.1111/j.1528-1157.1987.tb04218.x 3582291

[B14] Haerer AF Anderson DW Schoenberg BS Survey of major neurologic disorders in a biracial United States population: the Copiah County Study South Med J 1987 80 3 339 343 10.1097/00007611-198703000-00016 3824020

[B15] Schoenberg BS Recent studies of the epidemiology of epilepsy in developing countries: a coordinated program for prevention and control Epilepsia 1987 28 6 721 722 10.1111/j.1528-1157.1987.tb03707.x 3691419

[B16] Meneghini F Rocca WA Anderson DW Grigoletto F Morgante L Reggio A Savettieri G Di Perri R Validating screening instruments for neuroepidemiologic surveys: experience in Sicily. Sicilian Neuro-Epidemiologic Study (SNES) Group J Clin Epidemiol 1992 45 4 319 331 10.1016/0895-4356(92)90033-J 1314889

[B17] Placencia M Shorvon SD Parades V Bimos C Sander JWA Suarez J Cascante SM Epileptic seizures in an Andean region of Ecuador: Incidence and prevalence and regional variation Brain 1992 115 771 782 10.1093/brain/115.3.771 1628201

[B18] Nicoletti A Reggio A Bartoloni A Failla G Sofia V Bartalesi F Roselli M Gamboa H Salazar E Osinaga R Paradisi F Tempera G Dumas M Hall AJ Prevalence of epilepsy in rural Bolivia: a door-to-door survey Neurology 1999 53 9 2064 2069 10.1212/WNL.53.9.2064 10599782

[B19] Borges MA Min LL Guerreiro CA Yacubian EM Cordeiro JA Tognola WA Borges AP Zanetta DM Urban prevalence of epilepsy: populational study in Sao Jose do Rio Preto, a medium-sized city in Brazil Arq Neuropsiquiatr 2004 62 2A 199 204 10.1590/S0004-282X2004000200002 15235717

[B20] Melcon MO Kochen S Vergara RH Prevalence and clinical features of epilepsy in Argentina. A community-based study Neuroepidemiology 2007 28 1 8 15 10.1159/000097850 17164564

[B21] Noronha AL Borges MA Marques LH Zanetta DM Fernandes PT de Boer H Espindola J Miranda CT Prilipko L Bell GS Sander JW Li LM Prevalence and pattern of epilepsy treatment in different socioeconomic classes in Brazil Epilepsia 2007 48 5 880 885 10.1111/j.1528-1167.2006.00974.x 17326788

[B22] WHO/IBE/ILAE Atlas: Epilepsy Care in the World 2005

[B23] Mung'ala-Odera V Newton CR Identifying children with neurological impairment and disability in resource-poor countries Child Care Health Dev 2007 33 3 249 256 10.1111/j.1365-2214.2006.00714.x 17439437

[B24] Kaiser C Asaba G Leichsenring M Kabagambe G High incidence of epilepsy related to onchocerciasis in West Uganda Epilepsy Res 1998 30 3 247 251 10.1016/S0920-1211(98)00007-2 9657652

[B25] Medina MT Duron R Ramirez F Prevalence of neurological disorders in Tegucigalpa: the Kennedy study Rev Med Hond 2003 71 8 17

[B26] Villaran MV Montano SM Gonzalvez G Moyano LM Chero JC Rodriguez S Gonzalez AE Pan W Tsang VC Gilman RH Garcia HH Epilepsy and neurocysticercosis: an incidence study in a Peruvian rural population Neuroepidemiology 2009 33 1 25 31 10.1159/000210019 19325247 PMC2826439

[B27] Ruckinger S Boneberger A Epidemiologic challenges in rare diseases Bundesgesundheitsblatt Gesundheitsforschung Gesundheitsschutz 2008 51 5 483 490 10.1007/s00103-008-0533-6 18696139

[B28] Edwards T Scott AG Munyoki G Mung’ala Odera V Chengo E Bauni E Kwasa T Sander JWAS Neville BG Newton CRJC Active convulsive elipepsy in a rural district of Kenya: A study of prevalence and possible risk factors Lancet Neurol 2008 7 50 56 10.1016/S1474-4422(07)70292-2 18068520 PMC4058896

[B29] Meinardi H Scott RA Reis R Sander JW The treatment gap in epilepsy: the current situation and ways forward Epilepsia 2001 42 1 136 149 11207798 10.1046/j.1528-1157.2001.32800.x

[B30] Burderer NMF Statistical Methodology I: Incorporating the prevalence of disease into sample size calculation for sensitivity and specificity Acad Emerg Med 1996 3 895 900 10.1111/j.1553-2712.1996.tb03538.x 8870764

[B31] Mbuba CK Abubakar A Odermatt P Newton CR Carter JA Development and validation of the Kilifi Stigma Scale for Epilepsy in Kenya Epilepsy Behav 2012 24 1 81 85 10.1016/j.yebeh.2012.02.019 22481043 PMC3359498

[B32] Carpio A Lisanti N Calle H Borrero I Torres ME Toral AM Vasquez I Validation of a questionnaire for epilepsy diagnosis in primary care services Rev Panam Salud Publica 2006 19 3 157 162 10.1590/S1020-49892006000300003 16640844

[B33] Ottman R Barker-Cummings C Leibson CL Vasoli VM Hauser WA Buchhalter JR Validation of a brief screening instrument for the ascertainment of epilepsy Epilepsia 2010 51 2 191 197 10.1111/j.1528-1167.2009.02274.x 19694790 PMC2844922

[B34] Kelvin EA Hesdorffer DC Bagiella E Andrews H Pedley TA Shih TT Leary L Thurman DJ Hauser WA Prevalence of self-reported epilepsy in a multiracial and multiethnic community in New York City Epilepsy Res 2007 77 2–3 141 150 18023147 10.1016/j.eplepsyres.2007.09.012

[B35] Beran RG Michelazzi J Hall L Tsimnadis P Loh S False-negative response rate in epidemiologic studies to define prevalence ratios of epilepsy Neuroepidemiology 1985 4 2 82 85 10.1159/000110218 3831785

