# Supplementary material for: The validation of a three-stage screening methodology for detecting active convulsive epilepsy in population-based studies in health and demographic surveillance systems
Source: Emerg Themes Epidemiol. 2012 Nov 21;9:8. doi: 10.1186/1742-7622-9-8 (PMC3549939; doi:10.1186/1742-7622-9-8)
Supplement: Additional file 1 — Stage I (SI) of the cross-sectional survey: (Census screen for convulsions). [file 1742-7622-9-8-S1.doc]

**Additional file 1: Stage I (SI) of the cross-sectional survey: (Census screen for convulsions)**

Q1: Do you have fits or has someone ever told you that you have fits?

Q2: Do you experience episodes in which your legs or arms have jerking movements or fall to the ground and lose consciousness?
