# Supplementary material for: The validation of a three-stage screening methodology for detecting active convulsive epilepsy in population-based studies in health and demographic surveillance systems
Source: Emerg Themes Epidemiol. 2012 Nov 21;9:8. doi: 10.1186/1742-7622-9-8 (PMC3549939; doi:10.1186/1742-7622-9-8)
Supplement: Additional file 2 — Stage II (SII) screening questions. [file 1742-7622-9-8-S2.doc]

## Additional file 2: Stage II (SII) screening questions

Q1. Have you ever had a fit?

Q2. Has someone ever told you that you have fits?

Q3. Have you ever been told that you have epilepsy or epileptic fits?

Q4. Have you ever had attacks in which you fall to the ground with loss of consciousness?

Q5. Have you ever fallen to the ground without a reason and experienced:

1. Twitching?
2. Shaking of the arms or legs without control?
3. Wetting yourself?
4. Biting of the tongue?

Q6. Have you ever been told by a doctor that you have epilepsy or epileptic fits?

## If Yes to any of the above 6 questions:

1. When did the seizures start?
2. When was the last seizure?
3. Have you ever had any seizure within the last **5** years?
4. If **Yes,** how many seizures have you had within the last one year?
5. Did **all** the seizures occur with a febrile illness?
6. Are you currently using any drugs for convulsions?
